# The impact of sports preferences on physical activity participation among college students: the mediating role of sports achievement emotions and exercise motivation

**DOI:** 10.3389/fpsyg.2025.1565998

**Published:** 2025-04-28

**Authors:** Yi Sun, Yang Zhao, Jie Yang

**Affiliations:** ^1^College of Basic Medicine, Jining Medical University, Jining, Shandong, China; ^2^Department of Chinese and History, City University of Hong Kong, Kowloon, Hong Kong SAR, China; ^3^School of Sport and Health, Shandong Sport University, Jinan, Shandong, China

**Keywords:** sports preference, positive sports achievement emotions, negative sports achievement emotions, exercise motivation, physical activity participation

## Abstract

**Background:**

College students commonly exhibit low levels of physical activity participation, which not only impacts physical health but also negatively affects mental well-being and academic performance. Although existing research has focused on the effects of sports preference, sports achievement emotions, and exercise motivation on physical activity participation, the interactive mechanisms among these three factors have yet to be systematically explored.

**Objective:**

This study aims to investigate how college students’ sports preferences, sports achievement emotions, and exercise motivation influence physical activity participation through mediating effects.

**Methods:**

A cross-sectional questionnaire survey was conducted online, recruiting 801 undergraduates (58.18% female). The study utilized a sports preference scale, a sports achievement emotion questionnaire, an exercise motivation scale, and the number of days per week of moderate-to-vigorous physical activity to measure the variables. After controlling for demographic characteristics, mediation effect analysis was performed using Stata 17.0.

**Results:**

Sports preferences (SP) had a significant positive direct effect on physical activity participation (PAP) among college students (*B* = 55.494, *p* < 0.001). Positive sports achievement emotions (PSAE) significantly mediated the relationship between SP and PAP (indirect effect = 5.644, *p* < 0.001). Exercise motivation (EM) also exhibited a significant mediating effect (indirect effect = 2.304, *p* < 0.001). Moreover, a positive correlation between PSAE and EM was observed, creating a chain mediation effect (SP → PSAE → EM → PAP), with an indirect effect of 1.424 (*p* < 0.001). These findings suggest that sports preferences not only directly enhance physical activity levels but also promote physical activity participation through a multi-level pathway involving positive emotions and exercise motivation.

**Conclusion:**

College students’ sports preferences significantly predict physical activity participation, with positive sports achievement emotions and exercise motivation playing crucial roles as mediators and chain mediators. Future research should incorporate longitudinal designs or intervention experiments and include more diverse evaluation indicators to further validate and extend the findings of this study.

## 1 Introduction

The participation rate of college students in physical activity remains notably low, representing a significant public health issue that requires urgent attention on a global scale (Elgaddal et al., 2022). Despite the widely recognized health benefits of regular physical exercise, many college students fail to meet the recommended levels of physical activity (Trost et al., 2002; Karaca et al., 2016). Research has shown that insufficient physical activity is closely linked not only to an increased risk of physical health issues (Sinha, 2020) but also to mental health concerns such as anxiety and depression, as well as a decline in academic performance (Mahindru et al., 2023; Wang and Li, 2022; Herbert et al., 2020). As such, the effective promotion of physical activity participation among college students, particularly through the influence of psychological factors such as motivation, emotions, and preferences, has become a central topic in sports psychology research.

Sports preference refers to an individual’s interest and inclination towards specific types of physical exercise. Existing studies suggest a strong correlation between the intensity of sports preference and individuals’ engagement in physical activities (Chen et al., 2022). Vallerand et al. (1997) observed that the stronger an individual’s preference for a particular sport, the more likely they are to participate in it. Zeng and Sun (2022) further demonstrated that college students who prefer team sports and aerobic exercises engage in physical activity more frequently than those who favor static exercises (e.g., yoga or Pilates), indicating that sports preferences not only affect participation frequency but also play a significant role in determining the type of exercise chosen (Box et al., 2019). Moreover, sports preference is influenced by various factors, including cultural background, gender differences, and personal exercise experiences (Kim et al., 2022; De Vries, 2020).

Sports achievement emotions and exercise motivation are critical psychological mechanisms that affect participation in physical activities (Jeong and Cheng, 2020). Sports achievement emotions are the emotional responses individuals experience during exercise as a result of achievement experiences (Puig and Vilanova, 2011). These emotions can be classified into positive and negative categories. Positive emotions, such as pride and satisfaction, enhance emotional self-regulation, thus promoting sustained engagement in physical activities, while negative emotions, including anxiety and frustration, may undermine self-efficacy and reduce exercise participation (Lonsdale et al., 2009). While much of the research has emphasized the positive influence of positive emotions on exercise motivation (Biddle et al., 1996), negative emotions may impede exercise motivation by influencing individuals’ attitudes toward physical activity and self-efficacy (Fierro-Suero et al., 2020; Simonton and Garn, 2019). Furthermore, emotional intelligence plays a vital role in regulating sports achievement emotions and sustaining exercise motivation (Li et al., 2009).

Exercise motivation, especially intrinsic motivation, serves as a key driver for engaging in physical activities. According to Self-Determination Theory (Deci and Ryan, 2000), intrinsic motivation is typically associated with an individual’s interest in and enjoyment of the activity itself, as well as the satisfaction derived from exercise, whereas extrinsic motivation relies more on external rewards or social pressures. Intrinsic motivation is particularly important for long-term engagement in physical activity, while extrinsic motivation tends to influence only short-term participation (Kilpatrick et al., 2005; Sun, 2006). Research has demonstrated that fostering intrinsic motivation can significantly enhance an individual’s involvement in exercise, with a particularly pronounced impact on long-term participation among college students (Markland and Tobin, 2004).

Although previous studies have examined the independent effects of sports preference, sports achievement emotions, and exercise motivation on physical activity participation, there remains a scarcity of research on the interactions among these three factors. Existing literature typically focuses on the impact of a single factor on sports participation, with few studies systematically investigating the interrelations among these factors. Additionally, the dual role of positive and negative emotions in sports achievement emotions has not been fully explored. Unlike previous research that often isolates psychological mechanisms, this study innovatively integrates the intensity of sports preferences, emotional valence, and types of motivation into a unified analytical framework.

In light of the above, and drawing on Self-Determination Theory (Gagné and Deci, 2005) and Achievement Motivation Theory (Roberts, 1982), this study proposes the following hypotheses:

*Hypothesis 1*: Sports preference has a positive effect on physical activity participation, meaning that the stronger an individual’s preference for exercise, the higher the likelihood and frequency of their engagement in physical activity.

*Hypothesis 2a*: Positive sports achievement emotions mediate the relationship between sports preference and physical activity participation, such that an individual’s preference for exercise enhances participation motivation through the activation of positive sports achievement emotions, thereby promoting physical activity engagement.

*Hypothesis 2b*: Negative sports achievement emotions mediate the relationship between sports preference and physical activity participation, such that an individual’s preference for exercise suppresses participation motivation through the elicitation of negative sports achievement emotions, thereby reducing physical activity engagement.

*Hypothesis 3*: Exercise motivation mediates the effect of sports preference on physical activity participation.

*Hypothesis 4a*: Positive sports achievement emotions and exercise motivation jointly mediate the relationship between sports preference and physical activity participation in a chain-like manner.

*Hypothesis 4b*: Negative sports achievement emotions and exercise motivation jointly mediate the relationship between sports preference and physical activity participation in a chain-like manner.

In contrast to prior studies that have solely verified the impact of preference type, this study, through Hypotheses 2a and 2b, uncovers for the first time the dual-path mechanism through which preference intensity influences participation behavior via emotional valence. This advances beyond the limitations of traditional single-valence emotion research. The proposed hypothesis framework advances existing theory in three key ways: (1) by overcoming the dichotomy of motivation, revealing the reshaping effect of preferences on motivation (Hypothesis 3); (2) by extending emotional classification theory, validating the mediating heterogeneity of emotional valence (Hypotheses 2a and 2b); and (3) by innovatively integrating the phase characteristics of achievement motivation with sustained motivation theory in an interdisciplinary framework (Hypotheses 4a and 4b).

**Figure 1:**
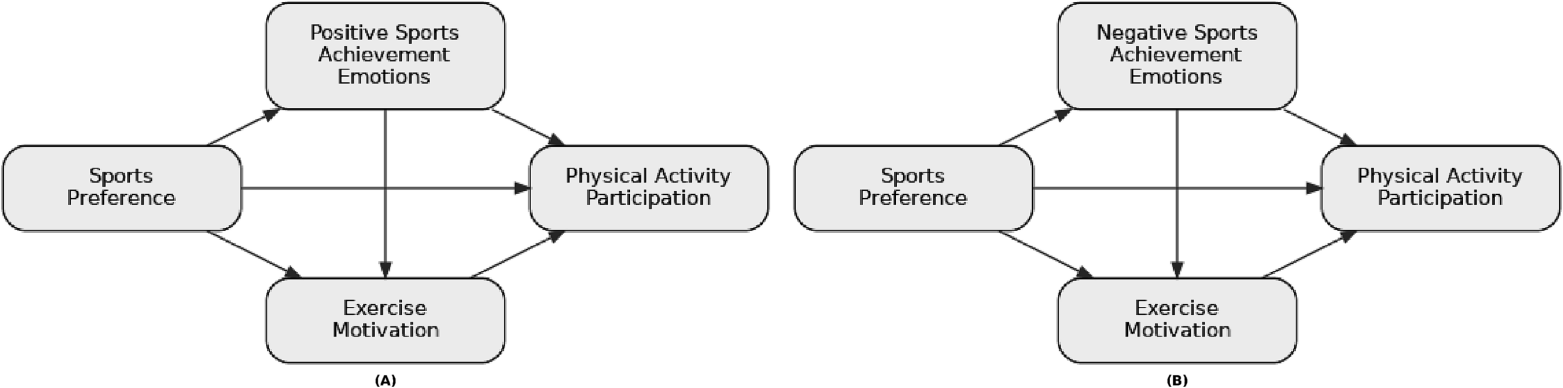
Research hypothesis model diagram.

The findings of this study not only provide a novel perspective for sports psychology theory but also offer practical guidance for university sports education and exercise interventions. The results will assist educators in considering students’ responses to both positive and negative emotions and their impact on exercise motivation when designing physical activity programs and interventions, thereby fostering sustained participation in physical activity. The hypothesized path model is depicted in Figure 1.

## 2 Methods

### 2.1 Participants

A total of 1,202 college students were recruited for this study, with data from 801 undergraduates meeting the criteria for analysis. Among the valid sample, 58.18% were female (*n* = 466) and 41.82% were male (*n* = 335). The participants’ ages ranged from 18 to 25 years (*M* = 20.94, SD = 1.33), with the average age for females being 20.85 years (SD = 1.34) and for males 21.18 years (SD = 1.29).

Of the sample, 34.21% were Han Chinese students (*n* = 274), while 65.79% were from minority ethnic groups (*n* = 527). The majority of participants came from rural areas (74.28%, *n* = 595), with third-year students constituting the largest group (54.06%, *n* = 433). Students majoring in engineering disciplines made up the highest proportion (34.71%, *n* = 278). Descriptive statistics for the variables are presented in Table 1.

**Table 1:** Descriptive statistics of study variables.

Variable	Mean (SD)	*n* (%)
Sports preference	62.76 (12.32)	
Sports achievement emotions	72.15 (13.90)	
Positive sports achievement emotions	28.29 (5.89)	
Negative sports achievement emotions	43.86 (12.77)	
Exercise motivation	57.94 (8.74)	
Physical activity participation	2.63 (1.81)	
Age (y)	20.98 (1.33)	
Gender		
Male		335 (41.82)
Female		466 (58.18)
Ethnicity		
Han ethnicity		274 (34.21)
Ethnic minorities		527 (65.79)
Home location		
Rural		595 (74.28)
Urban		206 (25.72)
Grade level		
Freshman		52 (6.49)
Sophomore		277 (34.58)
Junior		433 (54.06)
Senior		39 (4.87)
Major		
Humanities		266 (33.21)
Science		229 (28.59)
Engineering		278 (34.71)
Arts and sports		28 (3.5)

### 2.2 Procedure

This study was approved by the Ethics Committee of Ningxia Medical University (Approval No. JNMC-YX-2025-009) and strictly adhered to the ethical guidelines set forth in the Declaration of Helsinki. Data were collected via an electronic questionnaire on the Maike platform, with the study’s purpose clearly outlined in the recruitment information, emphasizing the voluntary nature of participation. The researchers stressed the importance of the survey’s independence and the authenticity of the data for the integrity of the study. All participants were recruited online and provided electronic informed consent prior to participation. To ensure data quality, two attention check questions were included in the questionnaire, and data from participants who either did not provide informed consent or failed the attention checks were excluded.

### 2.3 Instruments

#### 2.3.1 Sports preference

Sports preference was measured using the “College Students’ Sports Preference Scale” developed by Wang et al. (2020). The scale comprises three dimensions—social, subjective, and objective—and consists of 16 items. It aims to comprehensively assess students’ preferences for various sports activities. One example item is: “I think a person (such as a sports star or someone close to me) significantly influenced my preference for this sport.” Responses were recorded on a 7-point Likert scale, with 1 representing “Strongly Disagree” and 7 representing “Strongly Agree.” In this study, the Cronbach’s alpha for the sports preference scale was 0.894, indicating good internal consistency.

#### 2.3.2 Sports achievement emotions

This study utilized the Chinese version of the Sports Achievement Emotion Questionnaire (AEQ-PE) developed by Fierro-Suero et al. (2020) to measure sports achievement emotions (Tian et al., 2023). This scale includes 24 items that assess six categories of emotions: pride, joy, anger, anxiety, despair, and boredom. Positive sports achievement emotions include joy and pride, while negative emotions encompass anger, anxiety, despair, and boredom. The scale uses a 5-point Likert scale (1 = “Strongly Disagree,” 5 = “Strongly Agree”). In this study, the Cronbach’s alpha for the AEQ-PE was 0.921, demonstrating high internal consistency.

#### 2.3.3 Exercise motivation

Exercise motivation was assessed using the Chinese version of the “Physical Activity Motivation Scale” (MPAM-R) developed by Chen et al. (2008). This scale contains 15 items across five dimensions: health, competence, enjoyment, appearance, and social interaction. Responses were recorded on a 5-point Likert scale, with scores ranging from 1 (“Not at all”) to 5 (“Very Strongly”). The Cronbach’s alpha for the MPAM-R in this study was 0.940, indicating excellent internal consistency.

#### 2.3.4 Physical activity participation

Physical activity participation was measured by asking participants how many days per week they engage in moderate or vigorous physical activity for at least 30 min.

#### 2.3.5 Demographic characteristics

Demographic characteristics, including gender, age, ethnicity, hometown, grade level, and major, were also surveyed.

### 2.4 Statistical analysis

Data analysis was conducted using Stata 17.0, with a significance level set at 0.05. The analysis proceeded as follows: First, a Harman single-factor test was performed on all variables to detect common method bias. The results indicated that the variance explained by the first factor was only 21.03%, which is below the 40% threshold, suggesting a low risk of common method bias. Second, a distribution check revealed that the data were not normally distributed. According to previous studies (Hicks and Tingley, 2011), Stata’s mediation analysis method does not impose strict requirements regarding the normality of the data. The bootstrap method was employed for robust inferential testing, allowing for valid inferences without assuming a specific sampling distribution for the statistics. During the regression model validation process, this study examined potential multicollinearity issues. The variance inflation factors (VIF) for all variables were found to be less than 2.5, well below the critical value of 10, indicating that no significant multicollinearity issues were present among the variables.

Spearman’s correlation coefficient was used to examine the relationships between sports preference, sports achievement emotions, exercise motivation, and physical activity participation, in order to explore preliminary associations between these variables. For the mediation analysis, Stata’s mediation effect analysis tool was used, employing a bias-corrected bootstrapping method with 5,000 replications to compute the 95% confidence intervals for both direct and indirect effects.

## 3 Results

### 3.1 Correlation analysis

Table 2 presents the correlation coefficients between the variables. Sports preference was significantly positively correlated with exercise motivation (*r* = 0.363, *p* < 0.01), positive sports achievement emotions (*r* = 0.408, *p* < 0.01), and physical activity participation (*r* = 0.382, *p* < 0.01). Exercise motivation exhibited a strong positive correlation with positive sports achievement emotions (*r* = 0.503, *p* < 0.01) and a significant negative correlation with negative sports achievement emotions (*r* = −0.178, *p* < 0.01). Positive sports achievement emotions were also significantly positively correlated with physical activity participation (*r* = 0.175, *p* < 0.01), while negative sports achievement emotions showed a significant negative correlation with physical activity participation (*r* = −0.130, *p* < 0.01).

**Table 2:** Correlation matrix of observed variables.

Variable	1	2	3	4
1. Sports Preference	-			
2. Exercise Motivation	0.363	-		
3. Negative Sports Achievement Emotions	0.031	−0.178	-	
4. Positive Sports Achievement Emotions	0.408	0.503	−0.112	-
5. Physical Activity Participation	0.382	0.096	−0.130	0.175

### 3.2 Mediation analysis

#### 3.2.1 The mediating role of positive sports achievement emotions and exercise motivation

The results presented in Table 3 indicate that, after controlling for demographic characteristics, sports preference (SP) has a significant and positive direct effect on physical activity participation (PAP) among college students (*B* = 55.494, *p* < 0.001). This suggests that, all else being equal, a higher preference for sports among students is associated with greater physical activity participation, thereby confirming Hypothesis 1. This significant direct effect (*B* = 55.494) implies that for every unit increase in SP, the level of PAP increases by an average of 55.494 units, reflecting the strong influence of personal interests and preferences on behavioral choices.

**Table 3:** Mediating path analysis results of positive sports achievement emotions and exercise motivation.

Path	Meaning	*B*	95%CI		SE	*p*
SP → PAP	Direct Effect	55.494	44.253	66.736	5.735	<0.001
SP → PSAE	X → M	0.191	0.161	0.222	0.016	<0.001
SP → EM	X → M	0.172	0.126	0.218	0.023	<0.001
PSAE → EM	Indirect Effect	0.556	0.461	0.652	0.049	<0.001
PSAE → PAP	M → Y	29.503	5.123	53.884	12.439	0.018
EM → PAP	M → Y	13.382	−3.112	29.876	8.416	0.012
SP → PSAE → PAP	Indirect Effect	5.644	0.011	0.061	0.013	<0.001
SP → EM → PAP	Indirect Effect	2.304	0.000	0.035	0.009	<0.001
SP → PSAE → EM → PAP	Indirect Effect	1.424	0.000	0.021	0.005	<0.001

SP, sports preference; PAP, physical activity participation; PSAE, positive sports achievement emotions; EM, exercise motivation.

Positive sports achievement emotions (PSAE) play a significant mediating role in the relationship between sports preference and physical activity participation. Specifically, the regression coefficient for the effect of SP on PSAE was 0.191 (*p* < 0.001), and the path coefficient for PSAE on PAP was 29.503 (*p* = 0.018). Further analysis of the indirect effect revealed that SP indirectly influences PAP through PSAE, with an effect size of 5.644 (*p* < 0.001). This implies that stronger positive sports achievement emotions in students lead to a more pronounced effect of sports preference on physical activity participation, thus confirming Hypothesis 2a. This indirect effect accounted for approximately 8.9% of the total effect (direct effect + indirect effect), indicating that PSAE, as an emotional driver of sports activity, can transform students’ sports preferences into actual participation behaviors, creating a positive feedback loop.

Exercise motivation (EM) also mediates the relationship between sports preference and physical activity participation. The coefficient for SP on EM was 0.172 (*p* < 0.001), and the coefficient for EM on PAP was 13.382 (*p* = 0.012), resulting in an indirect effect (SP → EM → PAP) of 2.304 (*p* < 0.001). This indicates that sports preference can enhance exercise motivation, which, in turn, fosters greater physical activity participation, thereby supporting Hypothesis 3. This indirect effect accounted for 3.6% of the total effect. Although this proportion is relatively small, the statistical significance indicates that EM plays a significant role in transforming preference into actual behavior.

**Figure 2:**
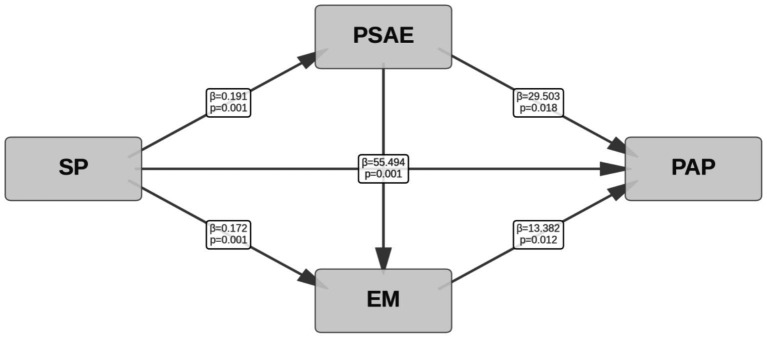
Mediating path diagram of positive sports achievement emotions and exercise motivation.

Importantly, a significant positive correlation was found between PSAE and EM (*B* = 0.556, *p* < 0.001), revealing a chain mediation effect (SP → PSAE → EM → PAP). The overall indirect effect for this pathway was 1.424 (*p* < 0.001), further supporting the multi-level mechanism involving sports preference, positive sports achievement emotions, and exercise motivation in influencing physical activity participation among college students. This finding thus confirms Hypothesis 4a. Although this chain mediation effect was relatively small in magnitude (accounting for 2.2% of the total effect), it theoretically reveals a complete psychological mechanism: SP first triggers PSAE, which in turn strengthen EM, ultimately leading to PAP. This finding has important implications for sports education practices, suggesting that fostering students’ positive sports achievement emotions may be a key factor in promoting sports participation (Figure 2).

#### 3.2.2 Mediating role of negative sports achievement emotions and exercise motivation

The results presented in Table 4 demonstrate that, after controlling for demographic characteristics, SP continues to have a significant and positive direct effect on PAP among college students (*B* = 57.848, *p* < 0.001). This indicates that as students’ preference for sports increases, their physical activity level also increases, thereby confirming Hypothesis 1. This direct effect (*B* = 57.848) is similar to the result in Table 3 (*B* = 55.494), further supporting the robust predictive effect of SP on PAP and suggesting that the influence of individual preferences on behavior remains consistent across different emotional models.

**Table 4:** Mediating path analysis results of negative sports achievement emotions and exercise motivation.

Path	Meaning	*B*	95%CI		SE	*p*
SP → PAP	Direct Effect	57.848	46.942	68.753	5.564	<0.001
SP → NSAE	X → M	0.035	−0.037	0.108	0.037	0.336
SP → EM	X → M	0.282	0.237	0.327	0.023	<0.001
NSAE → EM	Indirect Effect	−0.102	−0.145	−0.059	0.022	<0.001
NSAE → PAP	M → Y	−11.186	−20.941	1.432	4.977	0.025
EM → PAP	M → Y	2.994	−12.485	18.497	7.904	0.012
SP → NSAE → PAP	Indirect Effect	0.397	−0.003	0.012	0.004	0.062
SP → EM → PAP	Indirect Effect	0.845	0.000	0.032	0.012	0.016
SP → NSAE → EM → PAP	Indirect Effect	0.011	−0.001	0.000	0.000	0.051

SP, sports preference; PAP, physical activity participation; NSAE, negative sports achievement emotions; EM, exercise motivation.

However, the regression coefficient for SP predicting negative sports achievement emotions (NSAE) was 0.035 (*p* = 0.336), which was not statistically significant, suggesting that sports preference does not effectively alleviate the negative emotions students experience in sports settings. This finding is inconsistent with expectations, suggesting a potentially more complex relationship between SP and NSAE. It is possible that individuals with higher SP may also experience stress and failure emotions in competitive settings, reflecting the dual nature of emotional experiences in sports activities.

In the mediation path analysis, SP was found to significantly and positively predict EM (*B* = 0.282, *p* < 0.001), while NSAE had a significantly negative effect on EM (*B* = −0.102, *p* < 0.001), implying that as negative emotions increase, exercise motivation decreases. Further analysis revealed that the path coefficient for NSAE on PAP was −11.186 (*p* = 0.025), indicating that negative emotions may hinder physical activity participation. In contrast, the path coefficient for EM on PAP was 2.994 (*p* = 0.012), showing that exercise motivation positively promotes physical activity participation. Notably, although the negative effect of NSAE on PAP (*B* = −11.186) was smaller in absolute value compared to the promoting effect of positive sports achievement emotions (PSAE) (*B* = 29.503), it still reached statistical significance, revealing that NSAE may act as a psychological barrier to sports participation.

**Figure 3:**
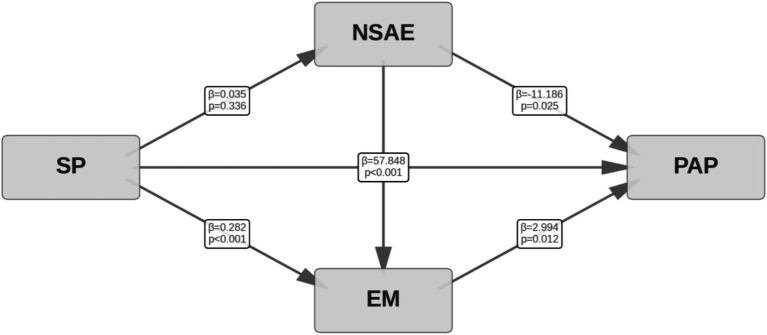
Mediating path diagram of negative sports achievement emotions and exercise motivation.

In the indirect effect analysis, SP’s indirect effect on PAP through NSAE was 0.397 (*p* = 0.062), which did not reach statistical significance, failing to support Hypothesis 2b. On the other hand, SP’s indirect effect on PAP through EM was 0.845 (*p* = 0.016), indicating that exercise motivation plays a significant mediating role in the relationship between sports preference and physical activity participation, thereby confirming Hypothesis 3. The chain mediation effect (SP → NSAE → EM → PAP) had an effect size of 0.011 (*p* = 0.051), which did not reach the traditional significance level, meaning Hypothesis 4b was not supported. These results suggest that, in the NSAE model, while EM as a mediator (*B* = 0.845) is significant, its effect size is only about 36.7% of the effect in the PSAE model (*B* = 2.304), reflecting the asymmetric influence of PSAE and NSAE on motivation and behavior (Figure 3).

## 4 Discussion

This study developed an integrative framework that incorporates sports preference (SP), positive and negative sports achievement emotions (PSAE/NSAE), and exercise motivation (EM) to explore the multiple pathways through which these factors influence physical activity participation (PAP) among college students. By conducting a chain mediation analysis, the study unveiled the underlying mechanisms at play. Based on cross-sectional survey data, the study yielded the following key findings:

First, the results confirmed the direct positive effect of sports preference on physical activity participation. In both the positive and negative sports achievement emotions models, SP demonstrated a significant positive predictive effect on PAP. This suggests that the more students are interested and engaged in sports, the more likely they are to invest time and effort in relevant training or activities. This finding aligns with the core principles of Self-Determination Theory (SDT) (Deci and Ryan, 2000), which asserts that intrinsic motivation, coupled with positive interactions with the external environment, can enhance behavioral engagement (Vansteenkiste et al., 2020), thereby fostering higher levels of physical activity participation (Deci and Ryan, 1985).

Second, the mediating effect of positive sports achievement emotions (PSAE) offers a novel perspective on understanding the mechanisms of sports participation. Unlike previous studies primarily focused on achievement motivation or subjective well-being (Swann et al., 2021), this study explores the unique role of achievement emotions (Guo et al., 2023). The findings reveal that positive emotional experiences in sports (e.g., pride, joy) not only enhance the effect of sports preference on physical activity participation but also generate a significant chain mediation effect (SP → PSAE → EM → PAP). This suggests that emotions act as both “lubricants” and “amplifiers” in student sports behavior (Bernstein and McNally, 2018): positive emotions stabilize sports preferences and amplify exercise motivation, leading to more sustained participation in sports (Kwan et al., 2017). This mechanism is consistent with the Broaden-and-Build Theory, which posits that positive emotions broaden cognitive-behavioral paradigms and help individuals accumulate psychological capital in broader contexts (Rhodes and Kate, 2015; Williams et al., 2008).

Third, the study confirms that exercise motivation (EM) plays a crucial mediating role in the transition from preference to behavior. Specifically, SP significantly boosts students’ EM, which in turn predicts increased engagement in physical activity. Both Self-Determination Theory and Achievement Goal Theory highlight that attractive sports activities are more likely to evoke intrinsic motivation, prompting individuals to set more challenging goals (Elliot and Hulleman, 2018). In this context, college students view sports as a pathway to self-improvement and enjoyment (Leyton-Román et al., 2021), thereby strengthening their resolve and planning for sustained involvement. Additionally, feedback mechanisms, including regular self-assessments, coach feedback (Carpentier and Mageau, 2016), and peer support (Scarapicchia et al., 2015), further reinforce positive attitudes toward sports and contribute to the formation of sustained physical activity.

However, negative sports achievement emotions (NSAE) present a more complex pattern of effects. The study found that frustration, anxiety, or dissatisfaction during exercise can weaken exercise motivation and engagement. Contrary to expectations, sports preference (SP) did not significantly predict NSAE levels. This finding warrants further discussion as it contrasts with Fredrickson’s (2001) theory of the asymmetry of positive and negative emotions and the findings of Campo et al. (2012). Existing research generally suggests that high SP should help alleviate negative emotions, but this study did not confirm this relationship, which may be attributed to several key factors: (1) while positive emotions are often directly triggered by success experiences, negative emotions can stem from multiple sources, including fear of failure, social comparison pressure, and unrealistic expectations (Hanin, 2007; Rice et al., 2016). This makes it difficult for a simple preference for sports to effectively regulate complex negative emotions. (2) For students with high SP, competitive settings may bring higher expectations of success alongside greater pressure from potential failure (Lazarus, 2000). Compared to individuals with lower SP, those with high SP may experience more intense emotional fluctuations when facing challenges or setbacks. (3) A high preference for sports does not necessarily imply strong emotional regulation skills (Fraser-Thomas and Côté, 2009). When effective emotion management strategies are lacking, even students passionate about sports may continue to experience negative emotions that can influence participation behavior.

Additionally, NSAE did not fully support the indirect effect path in SP → PAP (*B* = 0.397, *p* = 0.062), while in the positive sports achievement emotions (PSAE) model, the corresponding indirect effect was significant and had a larger effect size (*B* = 5.644, *p* < 0.001). This asymmetry further strengthens the differentiated roles of positive and negative emotions in the sports participation mechanism (Gross and John, 2019; Gardner et al., 2017). Regarding the mediating effect of exercise motivation (EM), the effect size in the NSAE model (*B* = 0.845) was only about 36.7% of that in the PSAE model (*B* = 2.304), suggesting that the positive emotional environment has a significantly stronger promoting effect on the formation of motivation and sustained behavior compared to the inhibiting effect of negative emotions.

This study constructed a multiple mediation model involving positive and negative sports achievement emotions and exercise motivation. It not only refines the emotional dimension classification but also comprehensively captures the differentiated impact of positive and negative emotions in sports contexts. Furthermore, it focuses on college students, a critical transitional group, making the findings highly valuable both theoretically and practically. Notably, the sports preference scale used in this study primarily measures an individual’s interest and preference for exercise, with limited focus on emotional regulation and coping mechanisms during exercise. This may explain why sports preference did not significantly predict negative sports achievement emotions. Based on the study’s results, university sports education practice can achieve applied transformation in five key directions: (1) strengthening emotion-oriented approaches; (2) implementing differentiated emotion management strategies; (3) systematically cultivating exercise motivation; (4) enhancing professional development for educators (improving emotional recognition skills, applying positive psychology, and fostering classroom emotional climate); and (5) optimizing policies (integrating emotional experiences into course evaluation metrics, increasing activity diversity, and establishing sports participation incentive mechanisms). Through these systematic measures, universities can build an emotionally supportive sports environment, effectively improving college students’ participation in physical activities and the benefits of exercise.

## 5 Limits and perspectives

This study has several limitations. The cross-sectional design makes it difficult to confirm causal relationships and the dynamic evolution of the effects. Future research should consider using longitudinal or experimental designs for more in-depth verification. Additionally, the study did not fully control for the effects of sport type, group characteristics, and contextual factors, and the intervention strategies and empirical evaluation of negative emotions require further refinement. The measurement tools primarily relied on self-reported questionnaires, and future research could integrate multi-source data (such as physiological indicators and behavioral observations) to enhance the validity of the results. Future studies might consider using more comprehensive measurement tools that not only assess the external manifestations of sports preference but also include emotional regulation abilities, psychological experiences during exercise, and coping mechanisms, thus providing a more accurate understanding of the complex relationship between sports preference and negative emotions.

## 6 Conclusion

This study empirically explores the multiple mechanisms through which sports preference, achievement emotions, and exercise motivation influence physical activity participation among college students. The findings reveal that sports preference not only directly enhances physical activity participation but also produces a significant chain mediation effect through positive sports achievement emotions and exercise motivation, thereby amplifying the positive impact of sports preference on sports behavior. While negative achievement emotions do suppress physical activity participation, their mediating role between sports preference and participation was not statistically supported. These results suggest that fostering positive interactions between sports preference, positive emotions, and intrinsic motivation may provide an effective pathway for promoting sustained physical activity participation among college students.

This study has several notable limitations. First, the cross-sectional research design cannot establish causal relationships between variables, as it only reflects associations at a specific point in time and is unable to reveal the dynamic evolution of these variables over time. Second, the study did not sufficiently control for the potential influence of factors such as the type of sport, individual trait differences, and specific exercise contexts on the research outcomes. Additionally, the study primarily relied on self-reported questionnaires to collect data, which may have been influenced by social desirability bias and individual cognitive differences, limiting the objectivity and accuracy of the results.

To validate and expand upon the current findings, future research should adopt more rigorous research designs. Longitudinal tracking studies are recommended to determine the temporal relationships and causal mechanisms between variables. Intervention experiments should be conducted to systematically assess the impact of specific strategies on emotional experiences, motivation development, and exercise participation. Moreover, combining objective measurement tools (such as physical activity data recorded by wearable devices) with qualitative research methods (such as in-depth interviews and observations) would provide more comprehensive and diverse evidence. Exploring the applicability of the model across different demographic characteristics, cultural backgrounds, and exercise environments would further enhance the generalizability and practical significance of the research findings.

## Data Availability

The raw data supporting the conclusions of this article will be made available by the authors, without undue reservation.
